# Discovery of a second‐site *nia2* mutation in the background of multiple *Arabidopsis* PIF‐related mutants containing the *pif3‐3* allele

**DOI:** 10.1111/nph.19344

**Published:** 2023-10-27

**Authors:** Zhe Ji, Eric J. Belfield, Shan Li, Xiangdong Fu, Nicholas P. Harberd

**Affiliations:** ^1^ Department of Biology University of Oxford Oxford OX1 3RB UK; ^2^ State Key Laboratory of Plant Cell and Chromosome Engineering, Institute of Genetics and Developmental Biology Chinese Academy of Sciences Beijing 100101 China; ^3^ National Key Laboratory of Crop Genetics & Germplasm Enhancement and Utilization Nanjing Agricultural University Nanjing 210095 China; ^4^ College of Life Sciences University of Chinese Academy of Sciences Beijing 100049 China; ^5^ New Cornerstone Science Laboratory Beijing 100101 China

**Keywords:** *Arabidopsis*, grafting, mutant, nitrate reductase, PHYTOCHROME‐INTERACTING FACTORs (PIFs)

Nitrogen (N) is one of the most needed mineral nutrients for plants due to its involvement in the biosynthesis of proteins, nucleic acids, and other essential cellular components such as chlorophyll and phytohormones (Crawford, 1995). Plants can extract N from the soil in a variety of forms, but nitrate (NO_3_^−^) represents the predominant source of N in most agricultural soils (Crawford & Forde, 2002). Nitrate is taken up by the root via NO_3_^−^ transporters belonging to the NITRATE TRANSPORTER1/PEPTIDE TRANSPORTER FAMILY (NRT1/NPF) and NRT2 families (Wang *et al*., 2012; Krapp *et al*., 2014). Subsequently, most NO_3_^−^ is translocated to shoot tissue to be reduced by nitrate reductase (NR) to nitrite (NO_2_^−^), which is converted into ammonium (NH_4_^+^) before being incorporated into the amino acid pool via the action of the glutamine synthase/glutamine oxoglutarate aminotransferase or glutamate synthase (GS/GOGAT) pathway (Campbell, 1999). As the enzyme catalysing the rate‐limiting step of NO_3_^−^ assimilation, and a major enzymatic source for the biosynthesis of the nitric oxide (NO) signalling molecule (Chamizo‐Ampudia *et al*., 2017; Khan *et al*., 2023), NR is under extensive regulation at both transcriptional and post‐transcriptional levels in response to various endogenous and environmental factors (e.g. Park *et al*., 2011; Lambeck *et al*., 2012; Konishi & Yanagisawa, 2013; Marchive *et al*., 2013; Creighton *et al*., 2017; Jamieson *et al*., 2022). Among them, light, in part through activating sugar assimilation, tightly regulates NR activity because photosynthesis provides the expensive reducing energy required for NO_3_^−^ reduction and needs to be coordinated with NO_3_^−^ metabolism to maintain the carbon (C)/N metabolic balance (Hoff *et al*., 1994; Baslam *et al*., 2020). However, the molecular mechanism of how light signalling directly modulates NO_3_^−^ metabolism remains largely elusive.

PHYTOCHROME‐INTERACTING FACTORs (PIFs) are basic helix–loop–helix (bHLH) transcription factors that negatively regulate light responses, and are degraded upon the activation of the phytochrome (phy) photoreceptors (Bae & Choi, 2008; Leivar & Quail, 2011; Xu *et al*., 2015). Moreover, accumulating evidence has demonstrated that PIFs are also targeted by other environmental (e.g. temperature) and developmental (e.g. phytohormones) signals to modulate plant responses, making them central hubs that integrate external stimuli to downstream biological activities (Balcerowicz, 2020; Sanchez *et al*., 2020). Therefore, we investigated whether PIFs additionally play a role in regulating NO_3_^−^ metabolism in the model plant *Arabidopsis thaliana*.

In this study, we used plant chlorate resistance to assess NO_3_^−^ metabolic capacity (see Supporting Information Notes S1 for Methods and materials). Chlorate is a NO_3_^−^ chemical analogue that can be taken up by NO_3_^−^ transporters and reduced by NR to chlorite, which is toxic to plants and causes leaf chlorosis (Wilkinson & Crawford, 1991; Tsay *et al*., 1993; Wang & Crawford, 1996). Under our growth conditions, *Arabidopsis nrt1.1* (lacking the dual‐affinity NO_3_^−^ transporter NRT1.1) and *chl3‐5* (lacking the major isoform of NR, NIA2, which accounts for 80–90% of total NR activity) mutants were resistant to chlorate toxicity (Fig. S1; Wilkinson & Crawford, 1991; Tsay *et al*., 1993), whereas the *nrt2.1 nrt2.2* (lacking two high‐affinity NO_3_^−^ transporters NRT2.1 and NRT2.2) and *nia1‐4* (lacking the minor NR isoform, NIA1) were not (Fig. S1). Next, given that PIFs have both shared and distinct regulatory roles (Jeong & Choi, 2013), we tested the chlorate response of the sextuple mutant *pqp6p7* (*pif1‐1 pif3‐3 pif4‐2 pif5‐3 pif6‐2 pif7‐1*), which lacks six of the eight PIFs, thus reducing the risk of potential PIF functional redundancy masking any mutant phenotype. Compared with wild‐type (WT), *pqp6p7* plants displayed significantly less leaf chlorosis caused by chlorate toxicity (Fig. 1a,b), suggesting that NO_3_^−^ metabolism was compromised in this mutant. Since the *pqp6p7* mutant was generated by crossing the quadruple *pifq* mutant (*pif1‐1 pif3‐3 pif4‐2 pif5‐3*) with *pif6‐2* and *pif7‐1*, we attempted to narrow down the causal mutation by treating these mutants with chlorate. Although a moderate level of chlorate resistance was observed for *pif7‐1*, only *pifq* mutant plants exhibited a comparable level of chlorate resistance to *pqp6p7* (Fig. 1c). Finally, we subjected the individual *pif* mutants that makeup *pifq* to chlorate treatment and found that *pif3‐3* exhibited chlorate resistance comparable with that of *pifq* and *pqp6p7* (Fig. 1d), suggesting that the resistance originated from there.

**Fig. 1:**
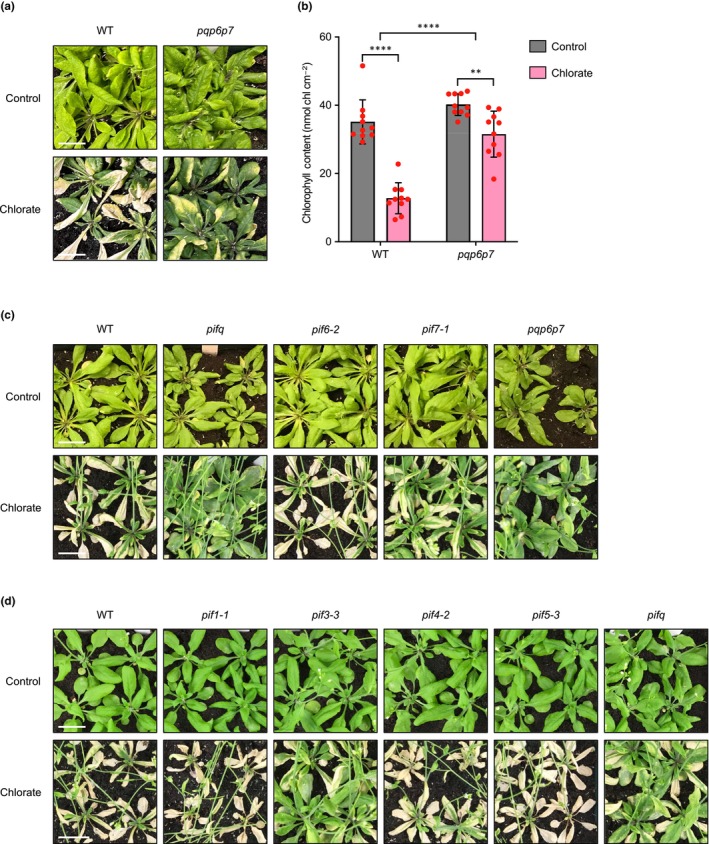
*pif3‐3* and higher‐order mutants containing the *pif3‐3* allele exhibit resistance to chlorate toxicity. (a) Chlorate responses of wild‐type (WT) and *pqp6p7* plants. Four‐week‐old plants grown on soil were treated with dH_2_O (control) or 1.5 mM chlorate every 5 d. When necessary, bolt stems were removed for ease of viewing. Bars, 2 cm. (b) Mean leaf chlorophyll contents, genotypes as shown, red dots indicate individual values (*n* = 10), error bars indicate SD. Statistical analysis was performed with two‐way analysis of variance (ANOVA): **, *P* ≤ 0.01; ****, *P* ≤ 0.0001. (c) Chlorate responses of WT, *pifq*, *pif6‐2*, *pif7‐1*, and *pqp6p7* plants. Four‐week‐old plants grown on soil were treated with dH_2_O (control) or 1.5 mM chlorate every 5 d. When necessary, bolt stems were removed for ease of viewing. Bars, 2 cm. (d) Chlorate responses of WT, *pif1‐1*, *pif3‐3*, *pif4‐2*, *pif5‐3*, and *pifq* plants. Four‐week‐old plants grown on soil were treated with dH_2_O (control) or 1.5 mM chlorate every 5 d. When necessary, bolt stems were removed for ease of viewing. Bars, 2 cm.

Since chlorate resistance can be attributed to a root‐dependent deficiency in NO_3_^−^/chlorate uptake, or a shoot‐dependent deficiency in NO_3_^−^/chlorate reduction, or both, we speculated that grafting might provide useful insight into the mechanisms of modified chlorate responses exhibited by mutants. As positive controls, we demonstrated that the graft chimaeras with WT shoot on *nrt1.1* root (WT/*nrt1.1*) and *chl3‐5* shoot on WT root (*chl3‐5*/WT) were resistant to chlorate, whereas the reciprocal graft chimaeras (*nrt1.1*/WT and WT/*chl3‐5*) were as sensitive to chlorate toxicity as the WT/WT controls (Fig. S2a,b). Subsequently, grafting was performed between WT and *pif3‐3* mutant plants. Among the resultant chimaeras, only those with a *pif3‐3* mutant shoot (*pif3‐3*/*pif3‐3* and *pif3‐3*/WT) were resistant to chlorate (Fig. 2a), suggesting a clear shoot‐dependency of the chlorate resistance originating from the *pif3‐3* mutant. Accordingly, compared with WT and the other *pif* mutants, mutants containing the *pif3‐3* allele exhibited a drastically reduced NR activity (Fig. 2b). Despite the lack of a detectable difference in the transcript levels of the two NR genes, *NIA1* and *NIA2* (Fig. S2c,d), the reduction in NR activity was reflected by a lower NR protein abundance in *pif3‐3* and *pifq* than that of WT (Fig. 2c). Cell‐free degradation assay showed that the rate of NR protein degradation was accelerated in *pif3‐3* compared with WT, indicating a reduction in NR protein stability (Fig. 2d).

**Fig. 2:**
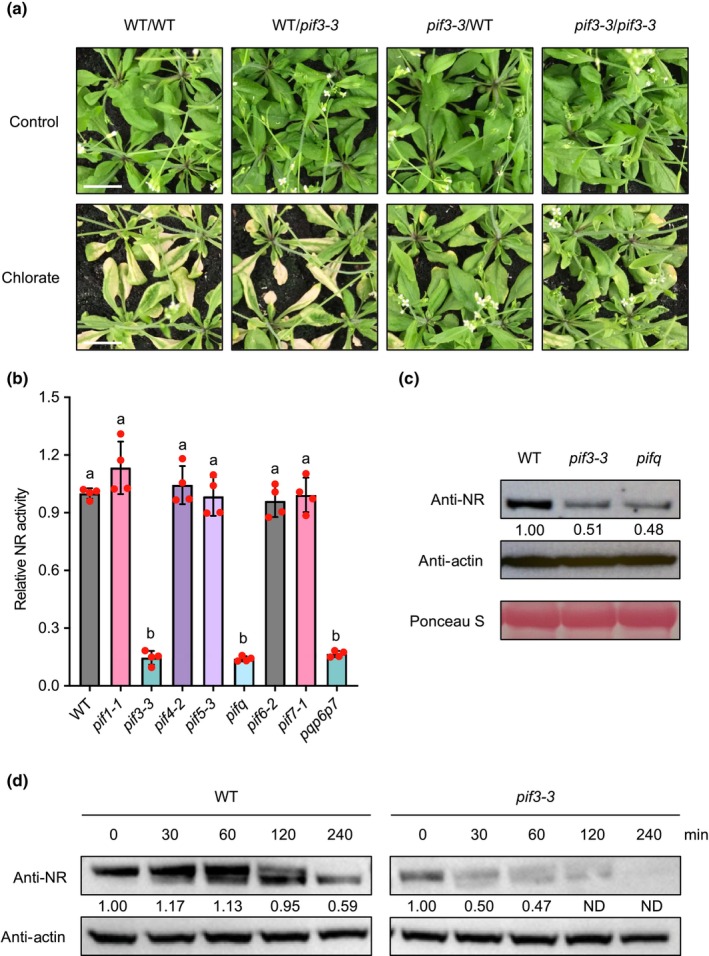
Shoot‐dependent deficiency in nitrate reductase (NR) activity underlies *pif3‐3*‐conferred chlorate resistance. (a) Chlorate responses of graft chimaeras made by exchanging shoots and roots of wild‐type (WT) and *pif3‐3* mutant plants. The graft chimaeras are labelled as shoot genotype/root genotype. All graft chimaeras were constructed at seedling stage before being transplanted to soil. Four‐week‐old plants were treated with dH_2_O (control) or 1.5 mM chlorate every 5 d. When necessary, bolt stems were removed for ease of viewing. Bars, 2 cm. (b) Mean relative shoot NR activity of WT and PHYTOCHROME‐INTERACTING FACTOR (PIF)‐related mutants. Red dots indicate individual values (*n* = 4), error bars indicate SD, and different letters (a, b) indicate significant differences (one‐way analysis of variance (ANOVA) with Tukey's test). (c) Abundance of immuno‐detected NR in WT, *pif3‐3*, and *pifq* plant extracts, quantified against Actin control (arbitrarily set at 1.00 for WT). Ponceau S staining serves as loading control. (d) Destruction rates of endogenous NR in WT and *pif3‐3* plant extracts, with immunodetectable NR quantified against Actin control (arbitrarily set at 1.00 for time point 0). ND, band not detected.

The *pif3‐3* allele was generated by fast neutron mutagenesis, which induced a 2.5‐kb deletion in the promoter region of *PIF3* that resulted in no detectable transcript of this gene (Fig. S3a; Monte *et al*., 2004; see Table S1 for primers used in this study). Two other *pif3* mutant alleles, *pif3‐1* and *pif3‐2*, contain T‐DNA insertions in the fourth intron of *PIF3* and produce truncated transcripts encoding a potential protein lacking a functional bHLH domain (Fig. S3a; Monte *et al*., 2004). Intriguingly, unlike *pif3‐3*, the *pif3‐1* and *pif3‐2* mutants exhibited similar chlorate responses and NR activities to WT (Figs 3a, S3b). To rule out the possibility that the truncated transcripts produced by *pif3‐1* and *pif3‐2* were functional in maintaining a WT level of NR activity, we generated our own *pif3* mutants using clustered regularly interspaced palindromic repeats (CRISPR)/CRISPR‐associated protein 9 (Cas9) that introduced frame shifts early in the gene (Fig. S3c), and confirmed that their NR activity was not affected by the mutations (Fig. 3b). Finally, we showed that the overexpression of *PIF3* in the *pif3‐3* mutant background led to an elongated hypocotyl phenotype characteristic of PIF over‐accumulation, but did not revert the repression of NR activity (Figs 3c, S3d,e). Collectively, we conclude that the observed low NR activity in *pif3‐3* is not due to the mutation in *PIF3 per se*, but rather to another mutation in the background, presumably caused by fast neutron bombardment.

**Fig. 3:**
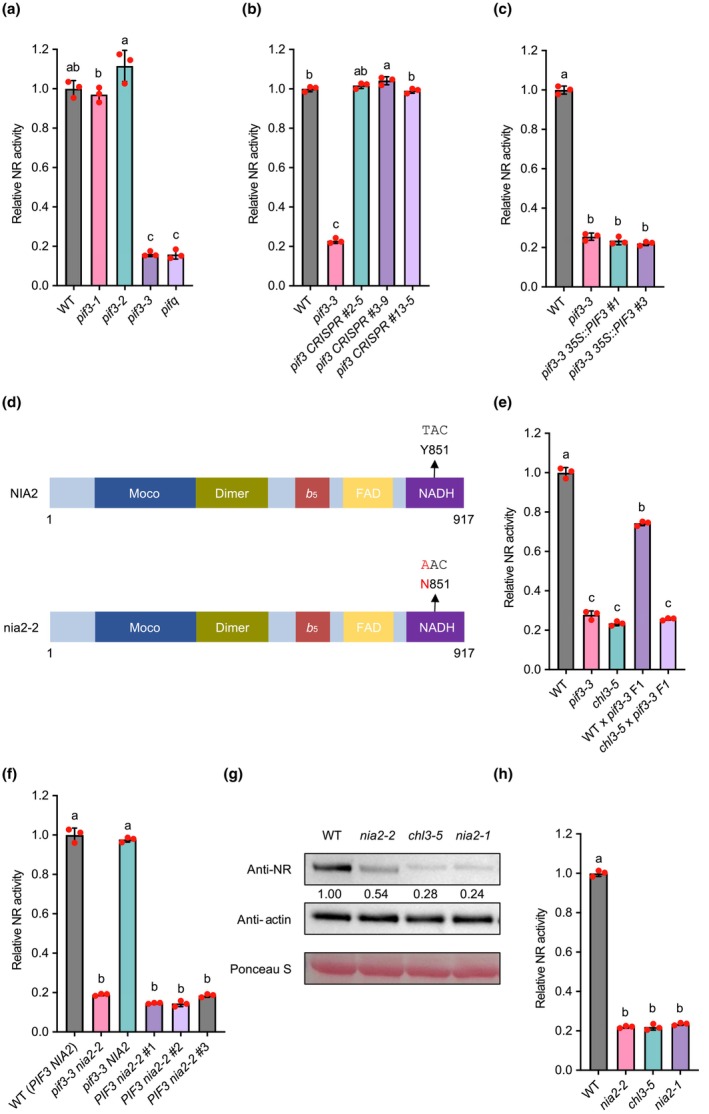
*nia2‐2* mutation in the background of *pif3‐3* is responsible for its compromised nitrate reductase (NR) activity. (a) Mean relative shoot NR activity of wild‐type (WT) and PHYTOCHROME‐INTERACTING FACTOR (PIF)‐related mutants. Red dots indicate individual values (*n* = 3), error bars indicate SD, and different letters (a–c) indicate significant differences (one‐way analysis of variance (ANOVA) with Tukey's test). (b) Mean relative shoot NR activity of WT, *pif3‐3*, and independent *pif3* mutants generated by clustered regularly interspaced palindromic repeats (CRISPR)/CRISPR‐associated protein 9 (Cas9)‐mediated genome editing. Red dots indicate individual values (*n* = 3), error bars indicate SD, and different letters (a–c) indicate significant differences (one‐way ANOVA with Tukey's test). (c) Mean relative shoot NR activity of WT, *pif3‐3*, and independent *pif3 35S::PIF3* Complementation lines. Red dots indicate individual values (*n* = 3), error bars indicate SD, and different letters (a, b) indicate significant differences (one‐way ANOVA with Tukey's test). (d) *nia2‐2* encodes a mutant nia2 protein with the Y851N amino acid substitution. Conserved subdomains of NIA2 are as indicated (Chamizo‐Ampudia *et al*., 2017). (e) Mean relative shoot NR activity of WT, *pif3‐3*, *chl3‐5*, and the F1 progeny made from crossing *pif3‐3* with either WT or *chl3‐5*. Red dots indicate individual values (*n* = 3), error bars indicate SD, and different letters (a–c) indicate significant differences (one‐way ANOVA with Tukey's test). (f) Mean relative shoot NR activity of WT (*PIF3 NIA2*), *pif3‐3 nia2‐2*, *pif3‐3 NIA2*, and independent *PIF3 nia2‐2* plants. Red dots indicate individual values (*n* = 3), error bars indicate SD, and different letters (a, b) indicate significant differences (one‐way ANOVA with Tukey's test). (g) Abundance of immuno‐detected NR in WT, *nia2‐2*, *chl3‐5*, and *nia2‐1* plant extracts, quantified against Actin control (arbitrarily set at 1.00 for WT). Ponceau S staining serves as loading control. (h) Mean relative shoot NR activity of WT, *nia2‐2*, *chl3‐5*, and *nia2‐1*. Red dots indicate individual values (*n* = 3), error bars indicate SD, and different letters (a, b) indicate significant differences (one‐way ANOVA with Tukey's test).

In order to identify the actual mutation in the background of *pif3‐3* that is responsible for its chlorate resistance and reduced NR activity, whole‐genome sequencing analysis was performed for the *pif3‐3* single mutant. We identified multiple single nucleotide variants (SNVs) and INDELs (insertions and deletions) present in the *pif3‐3* mutant (Datasets S1, S2). Among them, a SNV in the *NIA2* gene of *pif3‐3* led to a single amino acid substitution (Y851N) in the NADH‐binding domain of the NIA2 protein (Fig. 3d). To confirm that this mutant *nia2* allele (which we name *nia2‐2*) compromised NR activity, we performed allelism tests by crossing *pif3‐3* (with *nia2‐2* in the background) with known *nia2* loss‐of‐function mutants, *chl3‐5* (deletion of the entire *NIA2* gene) and *nia2‐1* (T‐DNA insertion *nia2* mutant), respectively. The F1 progeny from these crosses exhibited a similar level of NR activity as the parental *pif3‐3 nia2‐2* and *chl3‐5* or *nia2‐1* mutants (Figs 3e, S3f). On the contrary, F1 plants from crossing *pif3‐3 nia2‐2* with WT had an intermediate level of NR activity (Figs 3e, S3f), which is consistent with the fact that the *NIA2* gene is haploinsufficient (Wilkinson & Crawford, 1991). The F1 *PIF3*/*pif3‐3 NIA2*/*nia2‐2* heterozygous plants were allowed to self, and from the F2 population, we isolated individuals with only one of these two mutations, that is *pif3‐3 NIA2* and *PIF3 nia2‐2*. NR activity assay revealed that the *nia2‐2* mutation is solely responsible for the decreased NR activity in the original *pif3‐3* mutant and without it in the background, independent *pif3‐3 NIA2* lines isolated from the F2 population of a WT (*PIF3 NIA2*) × *pif3‐3 nia2‐2* cross do not have a detectably different NR activity compared with WT (*PIF3 NIA2*, Fig. 3f).

The Y851 residue locates within the C terminus domain of the NIA2 protein that binds to NADH, which provides the electron essential for NO_3_^−^ reduction (Campbell & Kinghorn, 1990). Furthermore, Y851 was found to be highly conserved in NIA orthologs across the plant kingdom from green algae to angiosperms, except for one of the two NIA homologues in *Physcomitrium patens*, whose corresponding site was occupied by a chemically similar phenylalanine (F) residue (Fig. S3g). Therefore, it is reasonable to speculate that Y851 at this position confers an important function, and substituting it with an asparagine (N) that is both structurally and chemically distinct from Y probably destabilises the protein (Fig. 2c,d). However, the protein abundance of NR in *nia2‐2* was still higher than that in *chl3‐5* or *nia2‐1*, even though the NR activity was indistinguishable between the three *nia2* mutants (Fig. 3g,h). Therefore, the Y851N substitution might additionally disrupt normal NR enzymatic activity.

Taken together, we report here the discovery of a hidden *nia2‐2* mutation in multiple mutants containing the *pif3‐3* allele and show that *nia2‐2* confers reduced NR activity, thus compromising NO_3_^−^ metabolism. This finding indirectly suggests that light‐mediated regulation of NR activity is unlikely to involve most of the PIF family proteins, although the mild chlorate resistance phenotype conferred by *pif7‐1* is worth exploring in future studies. The characterisation of the *nia2‐2* mutation also highlights the functional importance of Y851 in maintaining NR protein stability and enzymatic activity and provides a valuable mutant resource for studying NR functions. Lastly, we present a pipeline that can be referred to when studying a regulatory component of NO_3_^−^ metabolism. Specifically, we highlight the use of grafting as a novel and effective approach for spatially separating and distinguishing the effect on NO_3_^−^ uptake in the root and NO_3_^−^ assimilation in the shoot.

The *pif3‐3* mutant and higher‐order mutants containing the *pif3‐3* allele (e.g. *pifq* and *pqp6p7*) have been widely used to study PIF functions (e.g. Jiang *et al*., 2020; Bernula *et al*., 2021; Yoo *et al*., 2021; Piskurewicz *et al*., 2023; Sng *et al*., 2023). The identification of the hidden *nia2‐2* mutation revealed here might render it worthwhile re‐examining the alternative interpretation that some of the reported mutant phenotypes were due to the deficiency in N metabolism and/or NO production, especially for any studies investigating the crosstalk between the PIF and NO signalling pathways (Lozano‐Juste & León, 2011; Bai *et al*., 2014). We were initially surprised that the *nia2‐2* allele somehow evaded being eliminated through multiple rounds of crossing and persisted in the quadruple and sextuple *pif* mutants (especially considering that the original *pif3‐3* mutant was outcrossed twice with Col‐0 following its isolation, Monte *et al*., 2004). There is clearly not an absolute linkage between the *PIF3* and *NIA2* genes because we have successfully separated the two mutant alleles in this study. However, the fact that they reside on the same chromosome (PIF3: AT1G09530; NIA2: AT1G37130; Fig. S3h) suggests that they might be partially linked, and therefore, the mutant alleles may not assort completely independently during meiosis.

Forward genetics is a powerful approach for understanding gene function underlying a particular mutant phenotype induced by random mutagenesis in an unbiased manner that requires no prior knowledge about the gene of interest (Peters *et al*., 2003). However, random mutagenesis poses the risk of generating second‐site mutations that are responsible for the observed phenotype but are overlooked, which led to a plethora of recent publications unmasking these mutations in previously reported mutants (e.g. Bennett *et al*., 2006; Westphal *et al*., 2008; Enders *et al*., 2015; Gao *et al*., 2015; Kriegel *et al*., 2015; Wu *et al*., 2015; Yoshida *et al*., 2018; Vlad & Langdale, 2022; Yu *et al*., 2023). Therefore, we wish to remind the science community to not discount the possibility of a mutant material harbouring secondary mutations in the background, which seems to be particularly common for those generated from nonspecific mutagenesis. If possible, the use of other independent mutant alleles should always be included in their studies. Alternatively, complementation tests (using complete complementation gene constructs) can be performed to validate gene functions.

## Competing interests

None declared.

## Author contributions

NPH, EJB and ZJ conceived the project and designed the experiments. ZJ performed most of the experiments and wrote the manuscript. EJB analysed the whole‐genome sequencing data. SL assisted with the construction of plant transformants. XF provided creative input. All authors discussed the results and contributed to the manuscript.

## Supporting information


**Dataset S1** List of SNVs identified in *pif3‐3*.
**Dataset S2** List of INDELs identified in *pif3‐3*.

**Fig. S1** *Arabidopsis* mutants deficient in NO_3_^−^ uptake or NO_3_^−^ assimilation exhibit resistance to chlorate toxicity.
**Fig. S2** Validation of grafting as a means of understanding the mechanisms of modified chlorate responses exhibited by mutants.
**Fig. S3** *nia2‐2* mutation in the background of *pif3‐3* is responsible for its compromised NR activity.
**Notes S1** Methods and materials.
**Table S1** List of primers used in this study.
Please note: Wiley is not responsible for the content or functionality of any Supporting Information supplied by the authors. Any queries (other than missing material) should be directed to the *New Phytologist* Central Office.

## Data Availability

All data generated in this study are included in the main text and Supporting Information of this article. The Illumina DNA sequencing data files from this study have been submitted to the NCBI Sequence Read Archive (SRA; http://www.ncbi.nlm.nih.gov/sra) under accession no. SRR26298495.
