# Supplementary material for: Discovery of a second‐site nia2 mutation in the background of multiple Arabidopsis PIF‐related mutants containing the pif3‐3 allele
Source: New Phytol. 2023 Oct 27;241(1):17–23. doi: 10.1111/nph.19344 (PMC10952432; doi:10.1111/nph.19344)
Supplement: Supplementary file 2 — Fig. S1 Arabidopsis mutants deficient in NO3 − uptake or NO3 − assimilation exhibit resistance to chlorate toxicity. Fig. S2 Validation of grafting as a means of understanding the mechanisms of modified chlorate responses exhibited by mutants. Fig. S3 nia2‐2 mutation in the background of pif3‐3 is responsible for its compromised NR activity. Notes S1 Methods and materials. Table S1 List of primers used in this study. Please note: Wiley is not responsible for the content or functionality of any Supporting Information supplied by the authors. Any queries (other than missing material) should be directed to the New Phytologist Central Office. [file NPH-241-17-s001.pdf]

New Phytologist Supporting Information

Article title: Discovery of a second-site *nia2* mutation in the background of multiple *Arabidopsis* PIF-related mutants containing the *pif3-3* allele

Authors: Zhe Ji, Eric J. Belfield, Shan Li, Xiangdong Fu, and Nicholas P. Harberd

Article acceptance date: 25 September 2023

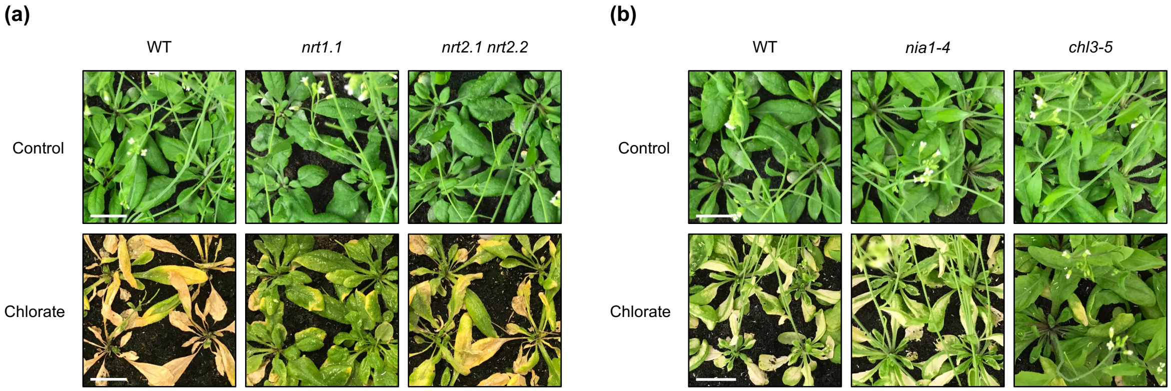

Fig. S1 *Arabidopsis* mutants deficient in NO_3^- uptake or NO_3^- assimilation exhibit resistance to chlorate toxicity. (a) Chlorate responses of four-week-old WT, *nrt1.1*, and *nrt2.1 nrt2.2* plants on soil. (b) Chlorate responses of four-week-old WT, *nia1-4*, and *chl3-5* plants on soil. When necessary, bolt stems were removed for ease of viewing. Scale bar = 2 cm.

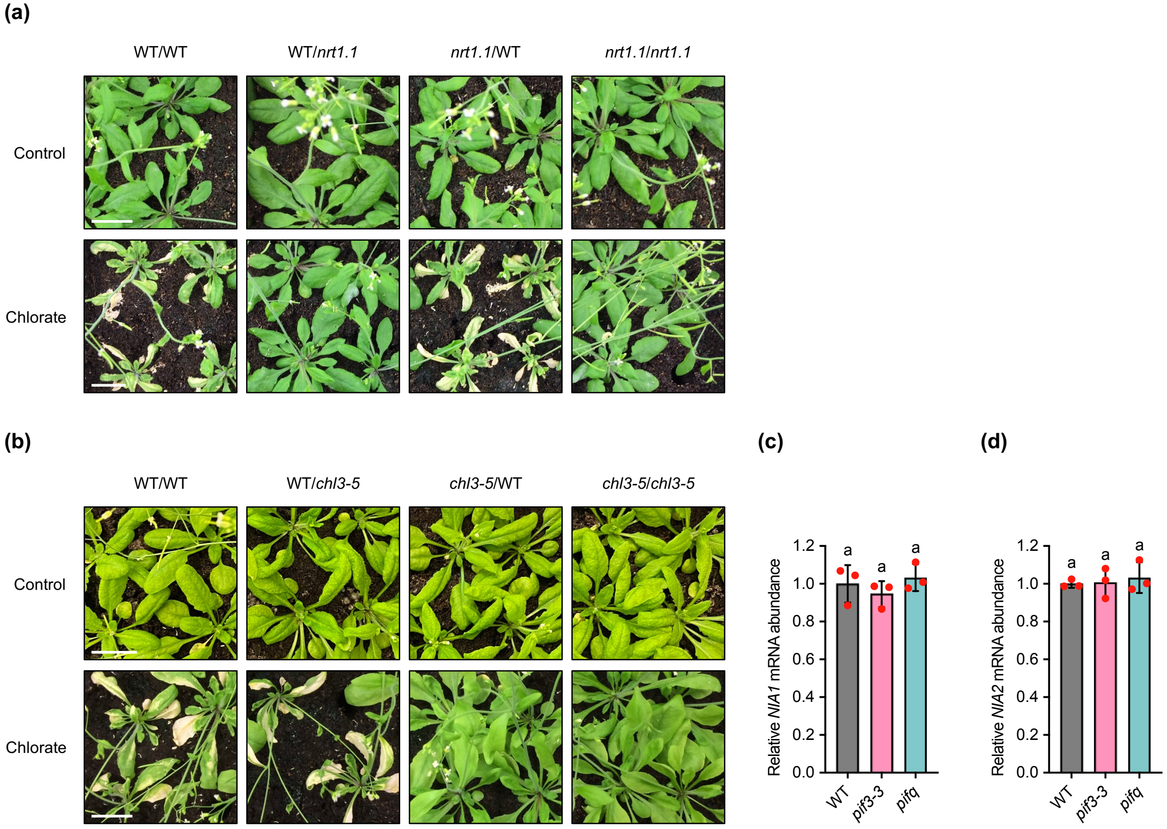

Fig. S2 Validation of grafting as a means of understanding the mechanisms of modified chlorate responses exhibited by mutants. (a) Chlorate responses of graft chimeras made by exchanging shoots and roots of WT and *nrt1.1* mutant plants. The graft chimeras are labelled as shoot genotype/root genotype. All graft chimeras were constructed at seedling stage before being transplanted to soil. Four-week-old plants were treated with dH_2O (control) or 1.5 mM chlorate every five days. When necessary, bolt stems were removed for ease of viewing. Scale bar = 2 cm. (b) Chlorate responses of graft chimeras made by exchanging shoots and roots of WT and *chl3-5* mutant plants. The graft chimeras are labelled as shoot genotype/root genotype. All graft chimeras were constructed at seedling stage before being transplanted to soil. Four-week-old plants were treated with dH_2O (control) or 1.5 mM chlorate every five days. When necessary, bolt stems were removed for ease of viewing. Scale bar = 2 cm. (c) Mean relative *NIA1* mRNA abundances in WT, *pif3-3*, and *pifq*. Red dots indicate individual values (n=3), error bars indicate standard deviation, no statistical difference was detected (one-way ANOVA with Tukey's test). (d) Mean relative *NIA2* mRNA abundances in WT, *pif3-3*, and *pifq*. Red dots indicate individual values (n=3), error bars indicate standard deviation, no statistical difference was detected (one-way ANOVA with Tukey's test).

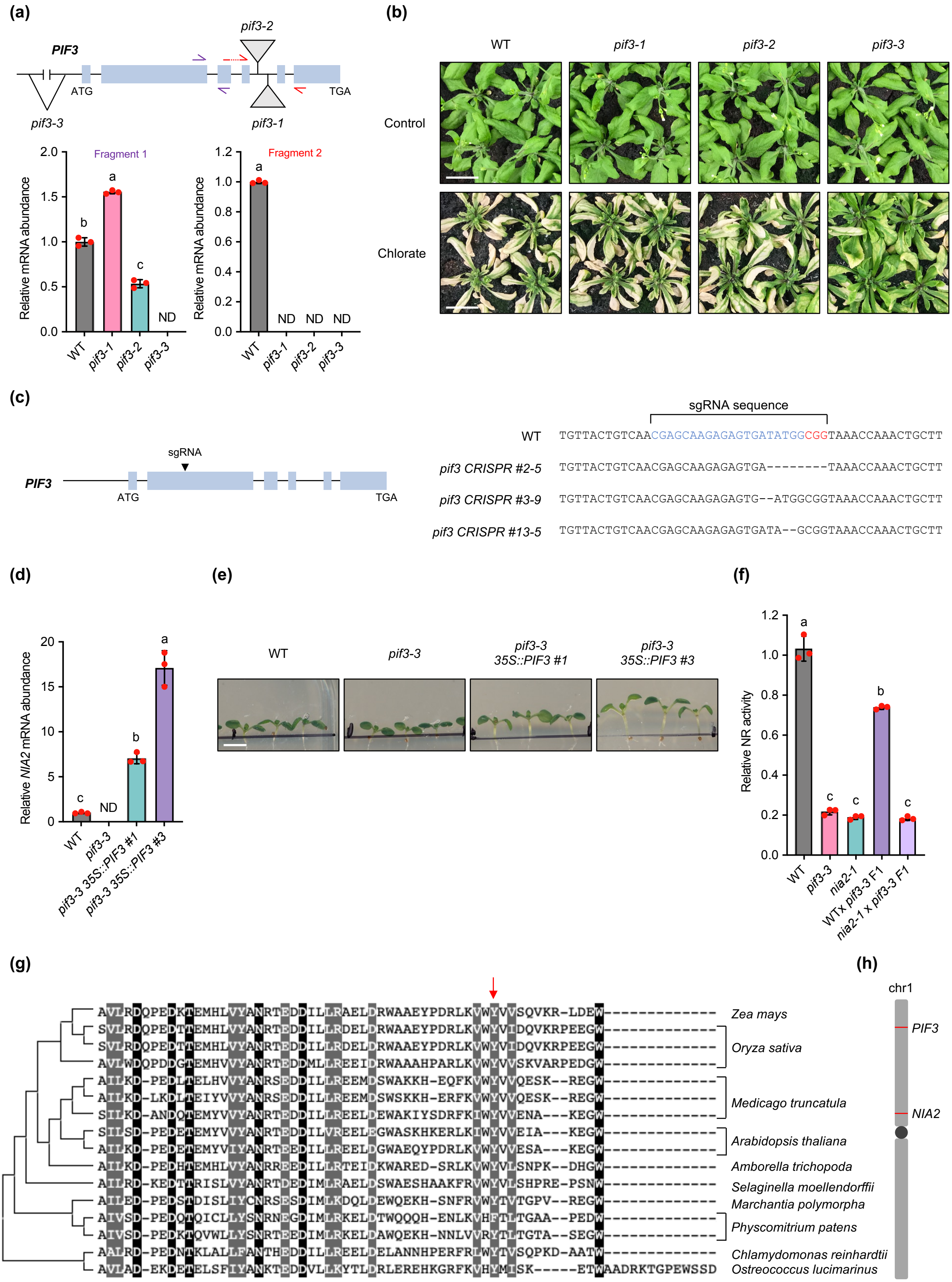
(a)
PIF3
ATG
TGA
pif3-3
pif3-2
pif3-1
Fragment 1
Fragment 2
Relative mRNA abundance
ND
WT
pif3-1
pif3-2
pif3-3
(b)
WT
pif3-1
pif3-2
pif3-3
Control
Chlorate
(c)
sgRNA
PIF3
ATG
TGA
sgRNA sequence
WT TGTTACTGTCAACGAGCAAGAGAGTGATATGGCGGTAAACCAAACTGCTT
pif3 CRISPR #2-5 TGTTACTGTCAACGAGCAAGAGAGTGA--------TAAACCAAACTGCTT
pif3 CRISPR #3-9 TGTTACTGTCAACGAGCAAGAGAGTG--ATGGCGGTAAACCAAACTGCTT
pif3 CRISPR #13-5 TGTTACTGTCAACGAGCAAGAGAGTGATA--GCGGTAAACCAAACTGCTT
(d)
Relative NIA2 mRNA abundance
WT
pif3-3
pif3-3 35S::PIF3 #1
pif3-3 35S::PIF3 #3
(e)
WT
pif3-3
pif3-3 35S::PIF3 #1
pif3-3 35S::PIF3 #3
(f)
Relative NR activity
WT
pif3-3
nia2-1
WTx pif3-3 F1
nia2-1 x pif3-3 F1
(g)
Zea mays
Oryza sativa
Medicago truncatula
Arabidopsis thaliana
Amborella trichopoda
Selaginella moellendorffii
Marchantia polymorpha
Physcomitrium patens
Chlamydomonas reinhardtii
Ostreococcus lucimarinus
(h)
chr1
PIF3
NIA2

Fig. S3 The *nia2-2* mutation in the background of *pif3-3* is responsible for its compromised NR activity. (a) Details of the *pif3-1*, *pif3-2*, and *pif3-3* alleles. The locations of T-DNA insertions (in the case of *pif3-1* and *pif3-2*) and fast neutron-induced deletion (in the case of *pif3-3*) are as indicated along the *PIF3* gene. The relative mRNA abundances of two fragments of *PIF3* (the primer pairs are labelled on the gene structure) in WT, *pif3-1*, *pif3-2*, and *pif3-3* are shown. Red dots indicate individual values (n=3), error bars indicate standard deviation, ND = not detected, different letters (a to c) indicate significant difference (one-way ANOVA with Tukey's test). (b) Chlorate responses of WT, *pif3-1*, *pif3-2*, and *pif3-3* plants. Four-week-old plants grown on soil were treated with dH_2O (control) or 1.5 mM chlorate every five days. When necessary, bolt stems were removed for ease of viewing. Scale bar = 2 cm. (c) Details of the *pif3* mutants generated in this study via CRISPR-Cas9-mediated genome editing. (d) Mean relative *PIF3* mRNA abundances in WT, *pif3-3*, and independent *pif3 35S::PIF3* complementation lines. Red dots indicate individual values (n=3), error bars indicate standard deviation, ND = not detected, different letters (a to c) indicate significant difference (one-way ANOVA with Tukey's test). (e) Seedling hypocotyl growth of WT, *pif3-3*, and independent *pif3 35S::PIF3* complementation lines. Scale bar = 5 mm. (f) Mean relative shoot NR activity of WT, *pif3-3*, *nia2-1*, and the F1 progeny made from crossing *pif3-3* with either WT or *nia2-1*. Red dots indicate individual values (n=3), error bars indicate standard deviation, different letters (a to c) indicate significant difference (one-way ANOVA with Tukey's test). (g) Phylogenetic analysis and multiple sequence alignment of NIA orthologues from diverse plant species. The tree was constructed in MEGA11 using the Maximum Likelihood method and JTT matrix-based model. The tree is drawn to scale, with branch lengths measured in the number of substitutions per site. Black highlights identical amino-acids, grey highlights amino acids that are similar. Red arrow highlights the Y851 site in AtNIA2. (h) Positions of the *PIF3* and *NIA2* genes on chromosome 1.

Note S1 Methods and Materials

Plant materials and growth conditions

All *Arabidopsis thaliana* plants used in this study were in the Columbia (Col) background. The following mutants were obtained from The European Arabidopsis Stock Centre (NASK): *pif1-1* (N66041), *pif3-1* (N530753), *pif3-2* (N581927), *pif3-3* (N66042), *pif4-2* (N66043), *pif5-3* (N66044), *pif6-2* (N667571), *pif7-1* (N68809), *pifq* (N66049), *pqp6p7* (N72486), *nrt1.1* (N597431), *nrt2.1 nrt2.2* (N535429), *nia1-4* (N684898), *chl3-5* (N2355), and *nia2-1* (N638297).

Arabidopsis seeds were sterilised with 75% ethanol for 10 minutes and germinated on half-strength Murashige & Skoog (MS) salt medium (pH 5.8) containing 0.5% sucrose, 0.5 g/L MES, and 1% agar at 22 °C in a 16-h light/8-h dark photoperiod (irradiance 120 μmol/m^2 per second). 10-day-old seedlings were transplanted to soil (ICL Levington Advanced F2 compost) and grown in controlled environment rooms (CERs) in the same environmental conditions as above.

Plasmid construction and *Arabidopsis* transformation

To generate *pif3* mutants by CRISPR/Cas9-mediated genome editing, primers containing the gRNA sequences were used to amplify the expression cassette on the pCBC-DT1DT2 vector. The PCR product was then cloned into linearised pHEE401E-EC2-Cas9-mCherry that was digested with BsaI, using In-Fusion Snap Assembly (TaKaRa). To make the *35S::FLAG-PIF3* overexpression construct, the coding sequence of PIF3 was amplified from cDNA and cloned into the pEarlyGate202 vector. The constructs were individually transformed into WT or *pif3-3 Arabidopsis* using the *Agrobacterium* (GV3101 strain)-mediated floral dip method (Zhang et al., 2006). Selection for edited T1 *pif3* mutant and T2 *Cas9*-free seeds were performed using a dissecting fluorescence microscope as described previously (Gao et al., 2016). Relevant primer sequences are listed in Table S1.

Chlorate toxicity assay and chlorophyll measurement

Four-week-old *Arabidopsis* plants growing on soil were treated with 1 L of 1.5 mM potassium chlorate ($KClO_3$) for 1 hour to reach maximum soil capacity, before the extra solution was

discarded. Control plants were treated with 1 L of dH_2O in the same manner. Chlorate/control treatment was repeated every five days for three times, or until a clear leaf chlorosis symptom was observed. Leaf chlorophyll content was used to quantify the extent of leaf chlorosis and was measured using a SPAD-502 meter (Konica-Minolta, Japan). The absolute chlorophyll concentration in nmol/mg fresh weight was calculated as previously described (Ling et al., 2011).

Arabidopsis hypocotyl graft

Arabidopsis seedling grafts were generated as previously described (Melnyk, 2017). Briefly, grafting was performed for seven-day-old seedlings in a sterile environment (in a laminar flow hood) under a stereomicroscope. Seedlings to be grafted were placed on pre-wet Hybond N membrane (GE Healthcare) and a clean horizontal cut was made on the hypocotyl at a position close to the shoot meristem. After exchanging the shoot with another seedling using a pair of forceps, graft chimeras were assembled by attaching the scion and rootstock tissues. Grafted seedlings were grown at 27 °C for five days to induce auxin production that facilitates graft recovery (Turnbull et al., 2002). Successfully grafted seedlings were transferred to soil and grown in CERs in the same environmental conditions as above. Throughout the experiment, any graft chimeras that developed adventitious roots were discarded.

NR activity assay

Total protein for NR activity assay was extracted with extraction buffer containing 50 mM KH_2PO_4 (pH 7.6), 1 mM EDTA, 5 mM DTT, 0.5% Triton and 1x HALT^{TM} Protease Inhibitor Cocktail (ThermoFisher), followed by two rounds of centrifugation at 20,000 g for 15 minutes. Protein concentration was determined using the Bradford Reagent (ThermoFisher) following the manufacturer's instructions. 200 µL of protein extract was added to 800 µL of reaction buffer consisted of 50 mM KH_2PO_4 (pH 7.6), 5 mM EDTA, 10 mM KNO_3 and 0.2 mM NADH, and incubated at 30 °C in the dark for 10 minutes. The reaction was stopped by adding an equal volume of 0.5 mM zinc acetate, followed by centrifugation at 16,000 g for 5 minutes. 500 µL of supernatant was added to 1 mL of colour development reagent made by mixing equal volumes of 1% sulfanilic acid in 3 M HCl and 0.1% *N*-(1-naphthyl)-ethylenediamine dihydrochloride. After incubation at room temperature for 30 minutes in

the dark, the absorbance of the reaction mixture at 540 nm (OD540) was measured using the Evolution 260 BIO UV-Visible Spectrophotometer, from which the NO_2^- concentration can be determined. NR activity was calculated as the concentration of NO_2^- produced per minute (nmol NO_2^- min^{-1}) normalised against sample protein concentration (mg ml^{-1}). Nitrite standard curve was made with serial dilutions of $NaNO_2$.

Reverse transcription quantitative PCR (RT-qPCR) analysis

Total RNA was extracted from seedlings with the TRIzol reagent (ThermoFisher) followed by DNase treatment using the DNA-free DNA removal kit (ThermoFisher) following the manufacturer's instructions. Full-length cDNA was then synthesised using the SuperScript IV Reverse Transcriptase (ThermoFisher) before being used for RT-qPCR analysis on the Applied Biosystem StepOnePlus Real-Time PCR System (Thermo Scientific) using the qPCRBIO SyGreen Mix Hi-Rox reagent (PCR Biosystems). RT-qPCR was performed with three biological replicates for each sample, and the transcript levels of target genes were normalised against that of the *Actin2* gene (At3g18780). Primers used for RT-qPCR are listed in Table S1.

Immunoblot analysis

Total protein from approximately 100 mg of plant material was extracted in extraction buffer consisting of 50 mM Tris–HCl (pH 7.5), 150 mM NaCl, 0.1% NP–40 detergent, 10% glycerol, 1 mM DTT and protease inhibitor cocktail (Roche). Protein samples were heated at 70 °C for 10 min before running on SDS–PAGE gels and blotted onto nitrocellulose membranes (VWR). The membrane was stained briefly until colour development in Ponceau S solution (Sigma-Aldrich), followed by de-staining in 0.1 M NaOH before blocking. NR and Actin proteins were detected using anti-NR (Agrisera, AS08 310) and anti-Actin (Agrisera, AS13 2640) antibodies, respectively. The protein bands were visualised on the iBright FL1500 Imaging System (ThermoFisher) and the band intensities were quantified with on-board image analysis.

Whole-genome sequencing analysis

DNA for whole-genome sequencing was extracted using the Plant/Fungi DNA Isolation Kit (Norgen) following the manufacturer's instructions, precipitated in 0.1 volume of 3 M NaOAc (pH 5.2) and 2.5 volumes of ethanol, and resuspended in 70% ethanol. This DNA was sequenced using 150-bp paired-end Illumina technology according to the manufacturer's

instructions at the Beijing Genomics Institute (BGI), China. Mutations were identified by aligning the sequencing result to the TAIR10 reference genome (Lunter & Goodson, 2011) as described previously (Gan et al., 2011). Single nucleotide variants (SNVs) and insertions and deletions (INDELs) in the *pif3-3* mutant were detected as described before (Belfield et al., 2018; Belfield et al., 2012; Jiang et al., 2014).

Phylogenetic analysis

Protein sequences of NIA orthologues from representative plant species were obtained from Phytozome (Goodstein et al., 2012) using AtNIA1 and AtNIA2 as queries for BLASTP. Multiple sequence alignment was performed using the T-Coffee alignment server (Di Tommaso et al., 2011) and phylogenetic trees were constructed in MEGA11 using the Maximum Likelihood method and JTT matrix-based model (Tamura et al., 2021).

Statistical data analysis

All statistical analyses (One-way ANOVA with Tukey's test and Two-way ANOVA) were performed using GraphPad Prism9. A *P*-value of less than 0.05 was considered to indicate statistical significance.

**Table S1** A list of primers used in this study.	
**Primers used to make transgene constructs**	
CRISPR_PIF3_F	CTAGAGTCGAAGTAGTGATTGGAGCAAGAGAGTGATATGGGTTTTAGAGCTAGAAATAGC
CRISPR_PIF3_R	TGCTATTTCTAGCTCTAAAACCAATCGTCGAAGAACCGGCCAATCTCTTAGTCGACTCTA
GW_PIF3_F	GGGGACAAGTTTGTACAAAAAAGCAGGCTCCATGCCTCTGTTTGAGCTTTTC
GW_PIF3_R	GGGGACCACTTTGTACAAGAAAGCTGGGTCCGACGATCCACAAAACTGAT
**Primers used in RT-qPCR**	
q_ACTIN2_F	CTGGATCGGTGGTTCCATTC
q_ACTIN2_R	CCTGGACCTGCCTCATCATAC
q_NIA1_F	AACGCAGGTACAGATTGC
q_NIA1_R	GGAAGAGTCGTAGCCAGT
q_NIA2_F	GGCGGCCTCTGTAGATAA
q_NIA2_R	AACGGGAGGTTTGTAAGA
q_PIF3_Fragment_1_F	GCCCATCCGAAAGTCCTTCA
q_PIF3_Fragment_1_R	CCAAACCCGTTCGAGATGGA
q_PIF3_Fragment_2_F	CTGAAAGGAGACGGCGTGATAG
q_PIF3_Fragment_2_R	CAGATAGTAACCAGACGCCATTGAC

References

Belfield, E. J., Ding, Z. J., Jamieson, F. J. C., Visscher, A. M., Zheng, S. J., Mithani, A., & Harberd, N. P. (2018). DNA mismatch repair preferentially protects genes from mutation. *Genome Res*, *28*(1), 66-74. https://doi.org/10.1101/gr.219303.116

Belfield, E. J., Gan, X., Mithani, A., Brown, C., Jiang, C., Franklin, K., Alvey, E., Wibowo, A., Jung, M., Bailey, K., Kalwani, S., Ragoussis, J., Mott, R., & Harberd, N. P. (2012). Genome-wide analysis of mutations in mutant lineages selected following fast-neutron irradiation mutagenesis of Arabidopsis thaliana. *Genome Res*, *22*(7), 1306-1315. https://doi.org/10.1101/gr.131474.111

Di Tommaso, P., Moretti, S., Xenarios, I., Orobitg, M., Montanyola, A., Chang, J. M., Taly, J. F., & Notredame, C. (2011). T-Coffee: a web server for the multiple sequence alignment of protein and RNA sequences using structural information and homology extension. *Nucleic Acids Res*, *39*(Web Server issue), W13-17. https://doi.org/10.1093/nar/gkr245

Gan, X., Stegle, O., Behr, J., Steffen, J. G., Drewe, P., Hildebrand, K. L., Lyngsoe, R., Schultheiss, S. J., Osborne, E. J., Sreedharan, V. T., Kahles, A., Bohnert, R., Jean, G., Derwent, P., Kersey, P., Belfield, E. J., Harberd, N. P., Kemen, E., Toomajian, C., . . . Mott, R. (2011). Multiple reference genomes and transcriptomes for Arabidopsis thaliana. *Nature*, *477*(7365), 419-423. https://doi.org/10.1038/nature10414

Gao, X., Chen, J., Dai, X., Zhang, D., & Zhao, Y. (2016). An Effective Strategy for Reliably Isolating Heritable and Cas9-Free Arabidopsis Mutants Generated by CRISPR/Cas9-Mediated Genome Editing. *Plant Physiol*, *171*(3), 1794-1800. https://doi.org/10.1104/pp.16.00663

Goodstein, D. M., Shu, S., Howson, R., Neupane, R., Hayes, R. D., Fazo, J., Mitros, T., Dirks, W., Hellsten, U., Putnam, N., & Rokhsar, D. S. (2012). Phytozome: a comparative platform for green plant genomics. *Nucleic Acids Res*, *40*(Database issue), D1178-1186. https://doi.org/10.1093/nar/gkr944

Jiang, C. F., Mithani, A., Belfield, E. J., Mott, R., Hurst, L. D., & Harberd, N. P. (2014). Environmentally responsive genome-wide accumulation of de novo Arabidopsis thaliana mutations and epimutations. *Genome Research*, *24*(11), 1821-1829. https://doi.org/10.1101/gr.177659.114

Ling, Q., Huang, W., & Jarvis, P. (2011). Use of a SPAD-502 meter to measure leaf chlorophyll concentration in Arabidopsis thaliana. *Photosynthesis research*, *107*(2), 209-214. https://doi.org/10.1007/s11120-010-9606-0

Lunter, G., & Goodson, M. (2011). Stampy: a statistical algorithm for sensitive and fast mapping of Illumina sequence reads. *Genome Res*, *21*(6), 936-939. https://doi.org/10.1101/gr.111120.110

Melnyk, C. W. (2017). Grafting with Arabidopsis thaliana. *Methods Mol Biol*, *1497*, 9-18. https://doi.org/10.1007/978-1-4939-6469-7_2

Tamura, K., Stecher, G., & Kumar, S. (2021). MEGA11: Molecular Evolutionary Genetics Analysis Version 11. *Mol Biol Evol*, *38*(7), 3022-3027. https://doi.org/10.1093/molbev/msab120

Turnbull, C. G., Booker, J. P., & Leyser, H. M. (2002). Micrografting techniques for testing long-distance signalling in Arabidopsis. *Plant J*, *32*(2). https://www.ncbi.nlm.nih.gov/pubmed/12383090

Zhang, X., Henriques, R., Lin, S. S., Niu, Q. W., & Chua, N. H. (2006). Agrobacterium-mediated transformation of Arabidopsis thaliana using the floral dip method. *Nat Protoc*, *1*(2), 641-646. https://doi.org/10.1038/nprot.2006.97
